# Editorial: Biopsychosocial Approaches to Transactional Sex

**DOI:** 10.3389/fpsyg.2021.729276

**Published:** 2021-07-20

**Authors:** Norbert Meskó, Béla Birkás, András Láng

**Affiliations:** ^1^Department for General and Evolutionary Psychology, Institute of Psychology, University of Pécs, Pécs, Hungary; ^2^Medical School, University of Pécs, Pécs, Hungary

**Keywords:** transactional sex, prostitution, biopsychosocial approaches, instrument development, sugar relationships

Transactional sex (e.g., prostitution, sugar relationships) is a specific form of relationship whereby material gains are exchanged for sex or companionship. It has presumably evolved, and has remained in existence in the modern day, as a result of a complex (bio-psycho-social) interaction between several factors (e.g., sex hormones, sexual desire for variety, social norms; see Meskó, 2014 for review). While researchers looking for proximate explanations of the phenomenon have focused mainly on the psychological, legal, and moral aspects of prostitution (e.g., Benoit et al., 2019), evolutionists have instead focused on the factors that lead to the emergence and persistence of prostitution (e.g., Dylewski and Prokop, 2019).

Where research focusing on proximal explanations of transactional sex is concerned, three basic paradigms are generally recognized (for an overview, see Meskó, 2014). The legal-moral paradigm underlines the assumption that using appropriate social controls, prostitution can be suppressed. The main focus of this paradigm is to find the most appropriate model that might be suitable for a particular type of social setting (e.g., size of settlement, population composition; Weitzer, 2000). However, it is often criticized for not providing a real solution to other problems associated with prostitution (e.g., sexually transmitted diseases, the criminalization of prostitution), as it essentially discriminates against prostitution and prostitutes. According to the gender equality paradigm, women's social opportunity depends on their ability to escape men's sexual repression, which can only be achieved if their sexual subordination to men ceases. Transactional sex may be the embodiment of this sex-based oppression, where sexuality is not an act between equal partners, but a business transaction. In this approach, the prostitute, while breaking social rules by selling her body, is also a victim (Bell, 1994). The paradigm of free choice focuses on the oppressive role of society in regulating prostitution. It suggests that engaging in transactional relationships is linked to the personal rights that women have, and the status of women in society can be diminished if such rights are limited (Alexander, 1998).

In this Research Topic, the paper of Ernst et al. describes the transactional sex phenomenon among university student in Germany within the paradigm of free choice. In their study, the knowledge and attitudes of non-sex worker students toward students who are sex workers in Berlin are examined. Furthermore, the motivation to participate in transactional sex, one's feelings related to sex work and its perceived risks were also assessed for students who have self-identified themselves as sex workers. Although there was no difference between students who were sex workers and those who were not in terms of happiness, the former still faced several of prejudices. The stigma and rejection they face have made it more difficult for them to seek and receive emotional support in their social environment. The authors point out that, if universities could play a role in emotionally supporting students who are engaging in sex work, it may help to empower these students and shape others' perspectives and choices about the sex industry in a more informed way.

Other articles in our Research Topic take a more evolutionary approach. Models regarding the ultimate mechanisms behind transactional sex are usually based on the sexually dimorphic parental inputs that might be apparent in terms of investment in one's offspring (for a review, see Meskó, 2014). Sex differences in reproductive investment in internally fertilized species also lead to sex differences in reproductive behavioral strategies (Trivers, 1972). In primates, a considerable amount of energy and resources is needed to produce offspring, and to protect and nurture them to their reproductive age. Human mate choice preferences also differ according to the level of reproductive/parental investment, and this might result in an increased sensitivity of women toward the resources of their potential mate. Men, who would invest less in the reproduction process and in terms of nurturing the offspring, are believed to place more emphasis on the physical fitness indicators of their potential mate. Based on cross-cultural analyses of mate choice preferences (Buss and Schmitt, 2019; Walter et al., 2020), human mating can be characterized by a combination of sexual availability and resources. Thus, men's preferences for women's reproductive capacity (e.g., youthfulness, fertility, health) and women's preferences for men's ability to possess material goods can be considered as adaptive traits.

In most of the studies without targeted sampling (e.g., evaluating the general population, not sex workers or prostitutes), <5% of participants reported being involved or having been involved in a transactional sexual relationship. Therefore, Birkás et al. develop a questionnaire assessing the openness to sugar relationships in younger women and men. In addition, Láng et al. develop another questionnaire to evaluate the attitudes of older men and women toward sugar relationships. Greater acceptance of sugar relationships in both age groups was associated with more unrestricted sociosexuality, a greater preference for playful love relationships, higher self-focused sexual motivation, and a more pronounced presence of Dark Triad (narcissism, psychopathy, and Machiavellianism) and borderline traits. These findings indicate that openness to transactional sex may be part of a broader mating strategy aimed at short-term resource maximization.

In a similar context, Davis et al. consider prostitution as a form of short-term mate choice and examine the motives of men who pay for sex. They found that the socially repugnant traits of the Dark Tetrad (narcissism, Machiavellianism, psychopathy, and sadism) are characteristic for these men, moreover, clients of female sex workers reported elevated desire for excitement and novel sexual experiences and to dominate sex workers and they tended to be unemotional toward prostitution.

Given the diversity of perspectives, the papers of this Research Topic might contribute to a more comprehensive framework of the phenomenon of transactional sex. The questionnaires reported in two of the papers could also be a starting point for further research on this topic.

## Author Contributions

NM wrote the first draft of the manuscript. BB and AL provided critical revision of the manuscript and important intellectual contributions. All authors contributed to the article and approved the submitted version.

## Conflict of Interest

The authors declare that the research was conducted in the absence of any commercial or financial relationships that could be construed as a potential conflict of interest.
